# Polystyrene Nanoplastics Induce Early Mitochondrial Dysfunction in H9c2 Cardiomyoblasts Without Substantial Cell Damage

**DOI:** 10.3390/antiox15070801

**Published:** 2026-06-26

**Authors:** Ming-Hung Shen, Pei-Hsuan Lu, Ting-Yu Tsai, Eddy Owaga, Yi-Sheng Tsai, Chia-Wen Chen, Rong-Hong Hsieh

**Affiliations:** 1 School of Nutrition and Health Sciences, College of Nutrition, Taipei Medical University, Taipei 11031, Taiwan; 2 Institute of Food Bioresources Technology, Dedan Kimathi University of Technology, Nyeri 10143, Kenya; 3 Research Center of Nutritional Medicine, College of Nutrition, Taipei Medical University, Taipei 11031, Taiwan

**Keywords:** polystyrene nanoplastics, nanoplastics, cardiomyoblasts, mitochondrial dysfunction, mitochondrial quality control, cellular uptake, environmental toxicology

## Abstract

Global plastic production has led to widespread contamination by micro- and nanoplastics, with polystyrene nanoplastics (PSNPs) increasingly being detected in human biological samples, including blood and cardiac tissue. Given the critical role of mitochondria in cardiac energy metabolism, this study investigated whether 100 nm PSNPs interact with mitochondria and affect mitochondrial function in H9c2 cardiomyoblasts. Cellular uptake and intracellular distribution were examined, followed by an evaluation of mitochondrial ultrastructure, intracellular and mitochondrial reactive oxygen species (ROS) production, mitochondrial membrane potential, mitochondrial dynamics and mitophagy-related gene expression, mitochondrial DNA copy number, and metabolic function. PSNPs were internalized but did not directly localize to mitochondria within 24 h. No significant cytotoxicity, increase in intracellular or mitochondrial ROS production, or alteration in basal metabolic activity was observed. However, PSNP exposure resulted in intracellular accumulation, an altered mitochondrial ultrastructure characterized by crista loosening and vacuole-like structural changes. These changes were accompanied by reduced mitochondrial membrane potential; the upregulation of mitochondrial dynamics-related genes, including optic atrophy 1 (*Opa1*) and dynamin-related protein 1 (*Drp1*); the suppression of PTEN-induced kinase 1 (*PINK1*)/Parkin RBR E3 ubiquitin protein ligase (*Parkin*)-mediated mitophagy-related genes; and decreased maximal respiratory capacity. Lactate production and the extracellular acidification rate remained unchanged, suggesting that compensatory glycolysis was not activated. These findings indicate that PSNP exposure induces early mitochondrial structural and functional alterations without substantial cell damage, suggesting a potential reduction in cardiac adaptive capacity under PSNP-induced stress conditions.

## 1. Introduction

Since the 1950s, global plastic production has increased dramatically, reaching nearly 400 million tons in 2022 [1]. The United Nations Environment Assembly (UNEA) has recognized plastic pollution as a critical issue in the fields of environmental and ecological sciences [2]. Common plastic products encountered in daily life are made from polystyrene (PS), polyethylene (PE), polyurethane (PU), polyethylene terephthalate (PET), polyvinyl chloride (PVC), polypropylene (PP) and polyamides (PAs) [3]. The environmental and biological degradation of plastic waste releases microplastics (MPs; 100 nm–5 mm) and nanoplastics (NPs; 1–100 nm) into oceans, soil, and air. These particles can enter the food chain and may be absorbed by humans through ingestion or inhalation, posing potential health risks [4].

PS is a widely used plastic material commonly found in food packaging, beverage containers, and electronic casings. Owing to its high production volume and low degradability, PS microplastics have emerged as major environmental pollutants [4]. Previous studies have mainly focused on the effects of polystyrene nanoplastics (PSNPs) on the digestive tract and intestines [5,6], and some reports also indicate potential pulmonary toxicity [7,8], suggesting that PSNPs can enter the human body through various exposure routes and exert toxic effects.

With increasing research, human evidence has gradually emerged. PSNPs have been detected in the bloodstream, ranking second among various types of plastic particles [9], and trace amounts have also been found in human cardiac tissue [10]. These findings indicate that PSNPs can enter systemic circulation and potentially reach distant organs, such as the heart. The long-term accumulation of PSNPs in the circulatory system may lead to endothelial dysfunction or abnormal cardiac function. Increasing research indicates that plastic pollution could become a new risk factor for cardiovascular diseases (CVDs) [11,12,13].

The heart is rich in mitochondria, which are essential for regulating energy metabolism and meeting the organ’s high energy demands. Given the critical role of mitochondria in energy production, their proper function is indispensable for maintaining overall cardiac health [14]. Beyond regulating cell growth and calcium homeostasis, mitochondria serve as central modulators of cellular redox balance. These organelles are a major source of intracellular reactive oxygen species (ROS), generated through electron leakage in the electron transport chain (ETC) during oxidative phosphorylation [15].

To maintain healthy mitochondria, mitochondrial quality control (MQC) mechanisms regulate their biogenesis, repair, and removal. Abnormal mitochondrial function or quality can lead to diseases such as cardiac fibrosis, arrhythmias, and heart failure [16].

The disruption of mitochondrial function by PSNPs may impair cellular energy metabolism and affect redox homeostasis, potentially compromising cardiomyoblast viability and cardiac health. The effects of 100 nm PSNPs on mitochondrial function, quality control, and redox-related processes in H9c2 cardiomyoblasts were explored. Given the central role of mitochondria in cellular redox regulation, we further examined PSNP uptake and intracellular distribution in relation to mitochondria, followed by an assessment of mitochondrial function, quality control pathways, and oxidative stress parameters.

## 2. Materials and Methods

### 2.1. Characterization of Polystyrene Nanoplastics

PSNPs (100 nm), including non-fluorescent (Cat. No. 00876-15) and green fluorescent-labeled particles (Cat. No. 17150-10), were obtained from Polysciences, Inc. The diameter and morphology of non-fluorescent PSNPs were characterized using transmission electron microscopy (TEM; HT-7700, Hitachi, Tokyo, Japan). The hydrodynamic particle size distribution was measured by dynamic light scattering (DLS), and zeta potential was determined by electrophoretic light scattering (ELS) using a Malvern Zetasizer Nano ZSP (Malvern Panalytical, Malvern, UK) equipped with a 10 mW He-Ne laser (633 nm). Measurements were performed at 25 °C in double-distilled water (ddH_2_O).

### 2.2. Cell Culture

H9c2(2-1) rat embryonic ventricular myoblasts (Bioresource Collection and Research Center (BCRC), No. 60096, Hsinchu, Taiwan) at passages 22–30 were used in this study. Cells were cultured in Dulbecco’s Modified Eagle Medium (DMEM; high glucose, 4.5 g/L; Thermo Fisher Scientific, Waltham, MA, USA; Cat. No. 12100046) supplemented with 4 mM L-glutamine (Thermo Fisher Scientific, Waltham, MA, USA; Cat. No. 25030081), 1.5 g/L sodium bicarbonate, 10% (*v*/*v*) fetal bovine serum (FBS; Hyclone, Logan, UT, USA; Cat. No. SH30396.03), and 1% penicillin/streptomycin (Biorion, Hsinchu, Taiwan; Cat. No. AACR0004B). Cultures were maintained at 37 °C in a humidified incubator with 5% CO_2_. When cells reached 80% confluence, they were subcultured. The medium was replaced every 2–3 days.

### 2.3. Preparation of Polystyrene Nanoplastics

Non-fluorescent PSNPs were used for all functional and bioenergetic assays, including cell viability, cellular ROS measurement, mitochondrial ROS detection, mitochondrial membrane potential analysis, Seahorse extracellular flux analysis, TEM, apoptosis analysis, lactate measurement, and quantitative polymerase chain reaction (q-PCR) assessment, whereas fluorescent PSNPs were used for imaging and internalization studies. Both were supplied as concentrated solutions and diluted with phosphate-buffered saline (PBS) to prepare stock solutions of 250, 500, 1000, and 1500 μg/mL, which were stored at 4 °C. For cell treatment, the stocks were further diluted 1:9 with fresh culture medium to obtain final concentrations of 25, 50, 100, and 150 μg/mL.

### 2.4. Experimental Design

H9c2 cells were assigned to five groups according to PSNP concentration: a control group (0 μg/mL PSNPs) and treatment groups exposed to 25, 50, 100, or 150 μg/mL of 100 nm PSNPs, designated as C, 25, 50, 100, and 150, respectively. After 24 h of exposure, subsequent analyses were performed.

### 2.5. Cell Viability

Cell viability was assessed using 3-(4,5-dimethylthiazol-2-yl)-2,5-diphenyltetrazolium bromide assay (MTT assay) (Sigma-Aldrich, Merck, St. Louis, MO, USA; Cat. No. M2128) and trypan blue exclusion assay. H9c2 cells (1 × 10^4^ cells/well) were seeded in 96-well plates and treated with PSNPs (0, 25, 50, 100, and 150 μg/mL) for 24 h. MTT solution (0.5 mg/mL in PBS) was added and incubated for 3 h at 37 °C in the dark, followed by the dissolution of formazan crystals in dimethyl sulfoxide (DMSO). Absorbance was measured at 570 nm using an Epoch 2 Microplate Spectrophotometer (Agilent BioTek, Winooski, VT, USA), with 690 nm used as the reference wavelength for background correction. For trypan blue exclusion, H9c2 cells (1 × 10^5^ per well) were seeded in 6-well plates and exposed to PSNPs (0, 25, 50, 100, and 150 μg/mL) for 24 h. Cell viability was then assessed using trypan blue exclusion with hemocytometer counting under an inverted microscope.

### 2.6. Quantification of Cellular Internalization of PSNPs

H9c2 cells (1 × 10^5^ cells/well) were seeded in 6-well plates and treated with various concentrations (0, 25, 50, 100, and 150 μg/mL) of PSNPs for 24 h. After treatment, cells were washed with PBS, collected, and centrifuged (1000 rpm, 3 min). The cell pellets were resuspended in PBS containing 5% (*v*/*v*) FBS and 0.1% 7-aminoactinomycin D (7-AAD, BioLegend, San Diego, CA, USA; Cat. No. 420403). Fluorescence signals were analyzed by flow cytometry using a BD FACSDiscover S8 cell sorter (BD Biosciences, Franklin Lakes, NJ, USA). PSNP uptake was detected based on the intrinsic fluorescence of polystyrene nanoplastics (λex/em = 441/485 nm) using 488 nm excitation and a 530/30 nm emission filter. Dead cells (7-AAD positive) and aggregates were excluded, and only single viable cells were analyzed. Real-time cell images were recorded during acquisition.

### 2.7. Localization of PSNPs

H9c2 cells (1 × 10^5^ cells/well) were cultured in 6-well confocal dishes and treated with fluorescent PSNPs at different concentrations (0, 25, 50, 100, and 150 μg/mL) for 24 h. Live-cell imaging was performed to evaluate PSNP intracellular localization using a Leica Stellaris 8 confocal microscope (Leica Microsystems, Wetzlar, Germany). Cell membranes were stained with Deep Red Dye (Abcam, Cambridge, UK; Cat. No. ab219942), nuclei with Nuclear Orange™ LCS1 (Cayman Chemical, Ann Arbor, MI, USA; Cat. No. 25174), and mitochondria with MitoTracker Red CMXRos (Invitrogen, Thermo Fisher Scientific, Waltham, MA, USA; Cat. No. 46752). Cells were incubated with each staining solution for 15 min at 37 °C in the dark, washed with PBS, and imaged in fresh medium. All staining procedures were performed according to the manufacturers’ instructions.

### 2.8. Colocalization Analysis of PSNPs

The colocalization of PSNPs with cellular structures was analyzed using Fiji software (ImageJ, version 1.54g, National Institutes of Health, USA) with the JACoP plugin [17]. Pearson’s correlation coefficient (PCC) was calculated to assess the pixel-wise correlation of fluorescence intensities between two channels, ranging from −1 (anticorrelation) to 1 (perfect correlation). Mander’s coefficients (M1 and M2) were used to quantify the fraction of overlapping signals between fluorescence channels. To correct for background fluorescence, threshold Mander’s coefficients (tM1 and tM2) were also calculated, ranging from 0 to 1, representing the intensity of colocalized signals between markers.

### 2.9. Cellular ROS Level

Cellular ROS levels were measured using a 2′,7′-dichlorofluorescin diacetate fluorescent probe (DCFDA; Cayman Chemical, Ann Arbor, MI, USA; Cat. No. 601520) and analyzed by an Invitrogen AttuneTM NxT Acoustic Focusing Cytometer (Thermo Fisher Scientific, Waltham, MA, USA). H9c2 cells (1 × 10^5^ cells/well) were seeded in 6-well plates and treated with various PSNP concentrations (0, 25, 50, 100, and 150 μg/mL) for 24 h. After treatment, cells were washed with PBS, trypsinized, collected by centrifugation, and resuspended in cell-based buffer. Cells were then stained with 10 μM DCFDA for 1 h at 37 °C in the dark, washed, and resuspended in cell-based buffer. Fluorescence was analyzed by flow cytometry using a 488 nm laser and a 530/30 nm emission filter and quantified as mean fluorescence intensity (MFI). Data were normalized to the control group. Flow cytometry data were collected from at least 10,000 events per sample. Debris and doublets were excluded based on forward scatter (FSC) and side scatter (SSC) profiles. Data analysis was performed using FlowJo software (version 10.9.0; BD Biosciences, Ashland, OR, USA).

### 2.10. Mitochondrial ROS Level

Mitochondrial ROS levels were measured using a Mitochondrial ROS Detection reagent (Cayman Chemical, Ann Arbor, MI, USA; Cat. No. 701600) and analyzed by an Invitrogen AttuneTM NxT Acoustic Focusing Cytometer (Thermo Fisher Scientific, Waltham, MA, USA). H9c2 cells (1 × 10^5^ cells/well) were seeded in 6-well plates and treated with different concentrations of PSNPs (0, 25, 50, 100, and 150 μg/mL) for 24 h. Cells were harvested by trypsinization, washed with PBS, and resuspended in cell-based buffer. Cells were then incubated with 0.5 μM pre-warmed mitochondrial ROS dye at 37 °C for 20 min in the dark. After washing with Hank’s Balanced Salt Solution (HBSS) twice, cells were resuspended in HBSS and immediately analyzed by a flow cytometer using a 488 nm laser and a 574/26 nm emission filter. Fluorescence intensity was quantified as MFI, and data were normalized to the control group. Flow cytometry data were collected from at least 10,000 events per sample. Debris and doublets were excluded based on FSC and SSC profiles. Data analysis was performed using FlowJo software.

### 2.11. Mitochondrial Membrane Potential

Mitochondrial membrane potential (ΔΨm) was assessed using tetramethylrhodamine ethyl ester (TMRE; Cayman Chemical, Ann Arbor, MI, USA; Cat. No. 701310) and analyzed by an Invitrogen AttuneTM NxT Acoustic Focusing Cytometer (Thermo Fisher Scientific, Waltham, MA, USA). H9c2 cells (1 × 10^5^ cells/well) were seeded in 6-well plates and treated with different concentrations of PSNPs (0, 25, 50, 100, and 150 μg/mL) for 24 h. Cells were harvested by trypsinization, washed with PBS, and incubated with 200 nM TMRE at 37 °C for 15 min in the dark. After washing with cell-based buffer, cells were resuspended and immediately analyzed by flow cytometry using a 488 nm laser and a 574/26 nm emission filter. Fluorescence intensity was quantified as MFI, and data were normalized to the control group. Flow cytometry data were collected from at least 10,000 events per sample. Debris and doublets were excluded based on FSC and SSC profiles. Data analysis was performed using FlowJo software.

### 2.12. Mitochondrial Oxygen Consumption Rate

Mitochondrial respiration and glycolytic function were evaluated using the Seahorse XF Cell Mito Stress Test Kit on a Seahorse XFe24 Extracellular Flux Analyzer (Agilent Technologies, Santa Clara, CA, USA). H9c2 cells (1 × 10^4^ cells/well) were seeded in Seahorse XFe24 microplates and treated with different concentrations of PSNPs (0, 25, 50, 100, and 150 μg/mL) for 24 h. Prior to the assay, cells were washed and incubated in Seahorse assay medium, which was prepared using sodium bicarbonate-free DMEM (high glucose) and adjusted to pH 7.4 on the day of the assay, for 1 h at 37 °C in a non-CO_2_ incubator. The oxygen consumption rate (OCR) and extracellular acidification rate (ECAR) were measured under basal conditions and following sequential injections of oligomycin (1 μM), CCCP (0.5 μM), and rotenone/antimycin A (0.5 μM). Basal respiration, maximal respiration, spare respiratory capacity, and glycolytic function were calculated according to the manufacturer’s instructions. OCR and ECAR values were normalized to total cellular protein content.

### 2.13. TEM Analysis of Mitochondrial Ultrastructure

For TEM analysis, H9c2 cells (1 × 10^5^ cells/well) were seeded in chamber slides and treated with different concentrations of PSNPs (0, 25, 50, 100, and 150 μg/mL) for 24 h. Cells were washed with PBS and fixed with a mixture of 2% paraformaldehyde and 2.5% glutaraldehyde. Fixed samples were processed for resin embedding and ultrathin sectioning at the Instrument Center of Taipei Medical University. Sections were examined using a TEM (HT-7700, Hitachi, Tokyo, Japan).

### 2.14. Apoptosis Analysis

Apoptosis was assessed using an FITC Annexin V Apoptosis Detection Kit with 7-AAD (BioLegend, San Diego, CA, USA; Cat. No. 640922) and analyzed by an Invitrogen AttuneTM NxT Acoustic Focusing Cytometer (Thermo Fisher Scientific, Waltham, MA, USA). H9c2 cells (1 × 10^5^ cells/well) were seeded in 6-well plates and treated with different concentrations of PSNPs (0, 25, 50, 100, and 150 μg/mL) for 24 h. Cells were harvested by trypsinization and resuspended in 100 μL Annexin V Binding Buffer containing 5 μL Annexin V-FITC, followed by incubation for 15 min. Cells were then stained with 4 μL 7-AAD in 400 μL Cell Staining Buffer for 5 min in the dark at room temperature. Samples were immediately analyzed by flow cytometry using a 488 nm excitation laser with a 530/30 nm emission filter for Annexin V-FITC and a 695/40 nm emission filter for 7-AAD. Apoptotic populations were quantified as early apoptotic (Annexin V^+^/7-AAD^−^), late apoptotic or necrotic (Annexin V^+^/7-AAD^+^), viable (Annexin V^−^/7-AAD^−^), and necrotic only (Annexin V^−^/7-AAD^+^) cells. Data were normalized to the control group. Flow cytometry data were collected from at least 10,000 events per sample. Debris and doublets were excluded based on FSC and SSC profiles. Data analysis was performed using Attune™ cytometric software (version 6.2.3; Thermo Fisher Scientific, Waltham, MA, USA).

### 2.15. Lactic Acid Assay

Lactic acid levels were measured to assess glycolytic activity. H9c2 cells (1 × 10^5^ cells/well) were seeded in 6-well plates and treated with different concentrations of PSNPs (0, 25, 50, 100, and 150 μg/mL) for 24 h. After treatment, culture supernatants were collected and analyzed using an L-lactic acid assay kit (Elabscience, China; Cat. No. E-BC-K044-M) according to the manufacturer’s instructions. Absorbance was measured with an Epoch 2 Microplate Spectrophotometer (Agilent BioTek, Winooski, VT, USA), and L-lactic acid concentrations were calculated from a standard curve.

### 2.16. Quantitative Polymerase Chain Reaction (q-PCR)

H9c2 cells (6 × 10^5^ cells/dish) were seeded in 10 cm dishes and treated with different concentrations of PSNPs (0, 25, 50, 100, and 150 μg/mL) for 24 h. Cells were washed with PBS, and the supernatant was removed. Thereafter, 1 mL of TRIzol (Ambion, Thermo Fisher Scientific, Waltham, MA, USA; Cat. No. 15596018) was added for total RNA extraction. A total of 40 ng RNA was reverse-transcribed into cDNA using the RevertAid First Strand cDNA Synthesis Kit (Thermo Fisher Scientific, Waltham, MA, USA; Cat. No. K1622), according to the manufacturer’s instructions. q-PCR was performed in a 25 μL reaction system using the DyNAmo ColorFlash SYBR Green qPCR Kit (Thermo Fisher Scientific, Waltham, MA, USA; Cat. No. F416L) on a QuantStudio 1 instrument (Thermo Fisher Scientific, Waltham, MA, USA). The q-PCR cycling conditions included an initial denaturation at 95 °C for 10 min, followed by 40 cycles of denaturation at 95 °C for 15 s, annealing at 60 °C for 30 s, and extension at 72 °C for 30 s. Relative gene expression was calculated using the comparative Ct method (2^−ΔΔCt^) and normalized to β-actin, which was used as the internal control based on previous reports of its stability in H9c2 cells. The primer sequences used in this study are listed in Table S1 and were designed based on previously published studies [18,19,20,21,22].

### 2.17. Mitochondrial DNA (mtDNA) Copy Number Analysis

NADH dehydrogenase subunit 1 (ND-1), encoded by mitochondrial DNA, was used as a marker for mitochondrial DNA copy number analysis [23]. A nuclear gene (β-actin) was used as the internal reference. H9c2 cells (6 × 10^5^ cells/dish) were seeded in 10 cm dishes and treated with different concentrations of PSNPs (0, 25, 50, 100, and 150 μg/mL) for 24 h. Cells were washed with PBS, and the supernatant was removed. Total DNA was extracted using the DNeasy Blood & Tissue Kit (QIAGEN, Hilden, Germany; Cat. No. 69506) according to the manufacturer’s instructions. mtDNA copy number was determined by qPCR. Relative mtDNA copy number was calculated using the ΔCt method by normalizing ND1 to the nuclear reference gene.

### 2.18. Statistical Analysis

The data were expressed as the mean ± standard deviation (SD). Statistical analysis was performed using GraphPad Prism version 9.0 (GraphPad Software, San Diego, CA, USA). A one-way analysis of variance (ANOVA) followed by Tukey’s post hoc test was used to analyze the differences between groups. A *p*-value of <0.05 was considered statistically significant.

## 3. Results

### 3.1. Characterization of PSNPs

**Figure 1:**
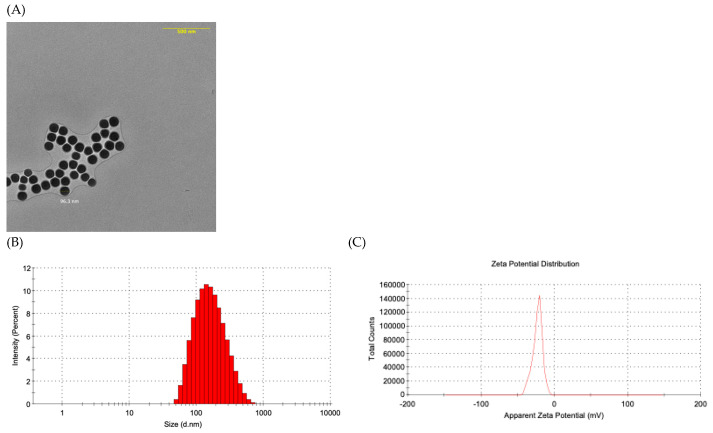
Characterization of PSNPs. (**A**) Representative TEM image of 100 nm PSNPs. Scale bar = 500 nm. (**B**) Hydrodynamic size distribution of PSNPs suspended in deionized water (ddH_2_O), determined by DLS. (**C**) Zeta potential distribution of PSNPs suspended in ddH_2_O measured by ELS.

The physicochemical properties of the PSNPs were characterized using TEM and DLS (Figure 1). TEM analysis showed that PSNPs were spherical with a mean particle diameter of 96.3 nm (Figure 1A). DLS measurements revealed a Z-average hydrodynamic diameter of 142.6 nm in ddH_2_O (Figure 1B). The zeta potential of PSNPs in ddH_2_O was −22.9 mV, indicating a negatively charged surface (Figure 1C).

### 3.2. PSNPs Exhibited Negligible Cytotoxicity on H9c2 Cardiomyoblasts

MTT and trypan blue exclusion-based cell counting assays were used to evaluate the cytotoxic effects of PSNPs on H9c2 cells and to determine appropriate treatment concentrations for subsequent experiments.

**Figure 2:**
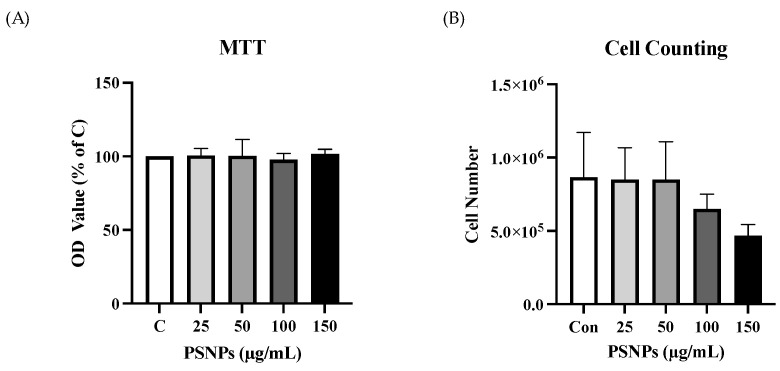
Cytotoxicity of PSNPs in H9c2 cells. H9c2 cells were treated with 100 nm PSNPs at various concentrations (0, 25, 50, 100, and 150 μg/mL) for 24 h. Cytotoxicity was analyzed by (**A**) MTT cell viability assay and (**B**) trypan blue exclusion assay. Data are expressed as mean ± SD (*n* = 6). Statistical analysis was performed using one-way ANOVA followed by Tukey’s post hoc test. Different letters indicate significant differences between groups (*p* < 0.05). C: 0 μg/mL as control group; 25: 25 μg/mL; 50: 50 μg/mL; 100: 100 μg/mL; 150: 150 μg/mL. PSNPs, polystyrene nanoplastics; MTT, methyl thiazolyl tetrazolium.

H9c2 cells were treated with PSNPs at concentrations of 0, 25, 50, 100 and 150 μg/mL for 24 h. Compared with the control group, no significant cytotoxic effects were observed in any treatment group, and no significant differences were detected among the PSNP-treated groups (Figure 2A,B).

### 3.3. Fluorescent PSNPs Were Taken up by H9c2 Cells in a Dose-Dependent Manner

H9c2 cells were treated with 0, 25, 50, 100, and 150 μg/mL of 100 nm fluorescent PSNPs for 24 h. Following pseudocolor processing during microscopic imaging, the cell membrane was labeled with Deep Red Dye, and the nucleus was counterstained with Nuclear Orange™ LCS1.

**Figure 3:**
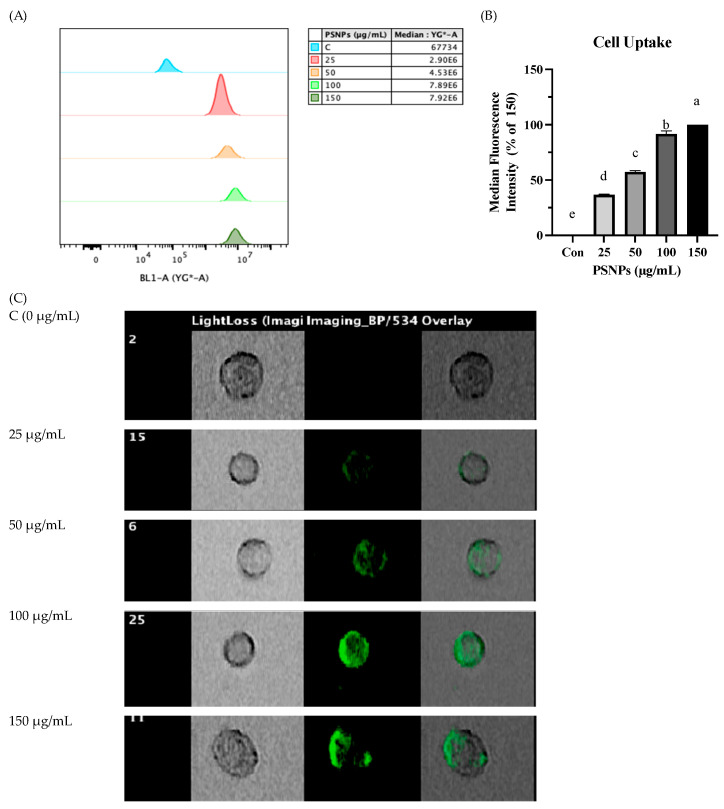
Cell uptake of fluorescent PSNPs in H9c2 cells. Cells were treated with 100 nm fluorescent PSNPs at various concentrations (0, 25, 50, 100, and 150 μg/mL) for 24 h, and then cell uptake level was analyzed by flow cytometry. (**A**) Data histogram was made by FlowJo. (**B**) Cell uptake level quantified and presented as MFI. (**C**) Real-time cell images captured during flow cytometry acquisition. Data are expressed as mean ± SD (*n* = 3). Statistical analysis was performed using one-way ANOVA followed by Tukey’s post hoc test. Different letters indicate significant differences between groups (*p* < 0.05). C: 0 μg/mL as control group; 25: 25 μg/mL; 50: 50 μg/mL; 100: 100 μg/mL; 150: 150 μg/mL. PSNPs, polystyrene nanoplastics; MFI, median fluorescence intensity.

Microscopic imaging showed that green fluorescent PSNPs were localized within the boundaries of the red-labeled cell membranes, indicating successful cellular uptake. Quantitative analysis and real-time imaging further demonstrated that the intracellular accumulation of PSNPs increased in a dose-dependent manner (Figure 3A–C).

### 3.4. Internalized PSNPs Remained Cytosolic Without Nucleus or Mitochondrial Translocation

**Figure 4:**
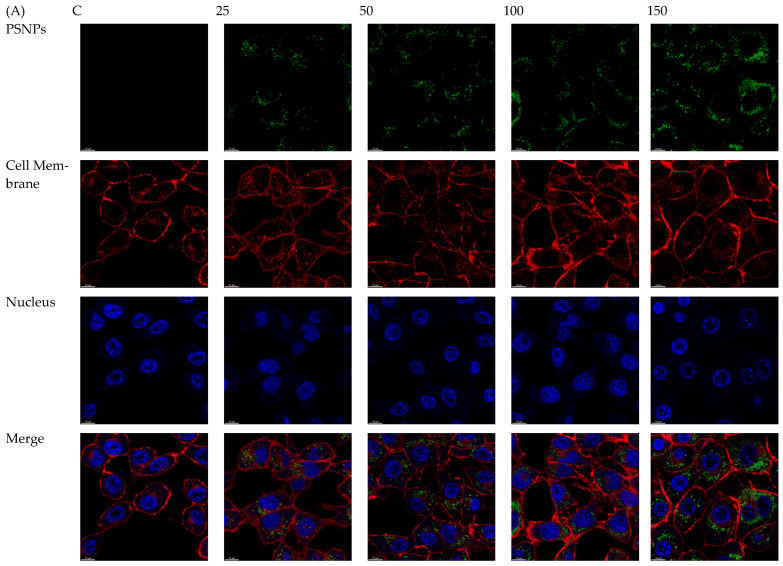

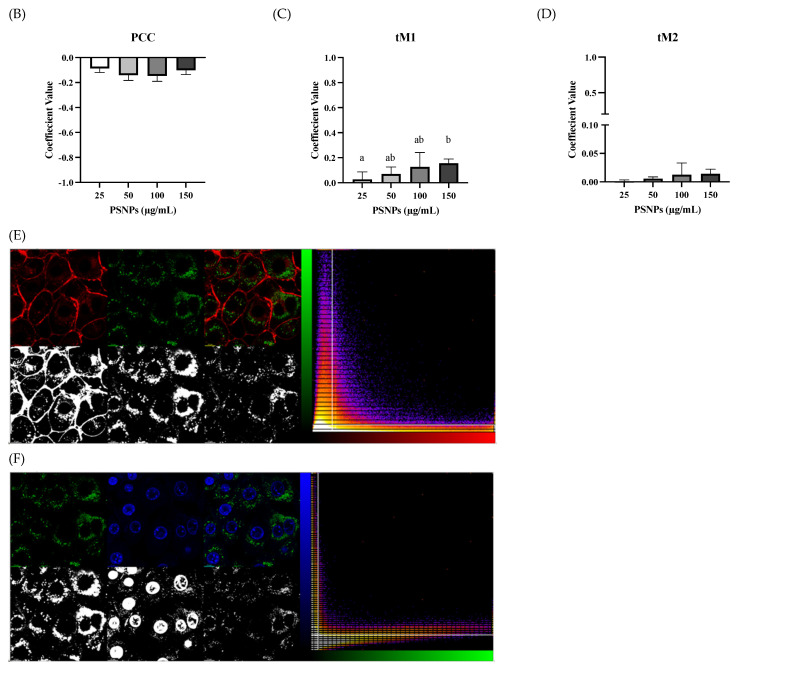
Intracellular localization and colocalization analysis of PSNPs in H9c2 cells. Cells were treated with 100 nm fluorescent PSNPs (green) at various concentrations (0, 25, 50, 100, and 150 μg/mL) for 24 h. (**A**) Confocal laser scanning microscopy (CLSM) images of subcellular location of PSNPs in H9c2 cells after exposure to different concentrations of PSNPs (green) for 24 h. Cell membranes were stained with Cytopainter Deep Red (Red), and nuclei were labeled with Nuclear Orange™ LCS1 (blue). Colocalization between nucleus and PSNPs is measured by (**B**) Pearson’s correlation coefficients (PCCs). (**C**,**D**) Threshold Mander’s correlation coefficients 1 and 2, respectively. (**E**,**F**) Colocalization fluorescence intensity scatter plots of 150 μg/mL group. (**E**) PSNPs versus nucleus fluorescence intensity correlation. (**F**) PSNPs versus cell membrane fluorescence intensity correlation. All plots were generated from same raw image. Data are expressed as mean ± SD (*n* = 6). Statistical analysis was performed using one-way ANOVA followed by Tukey’s post hoc test. Different letters indicate significant differences between groups (*p* < 0.05). C: 0 μg/mL as control group; 25: 25 μg/mL; 50: 50 μg/mL; 100: 100 μg/mL; 150: 150 μg/mL. PSNPs, polystyrene nanoplastics; PCC, Pearson’s correlation coefficient.

**Figure 5:**
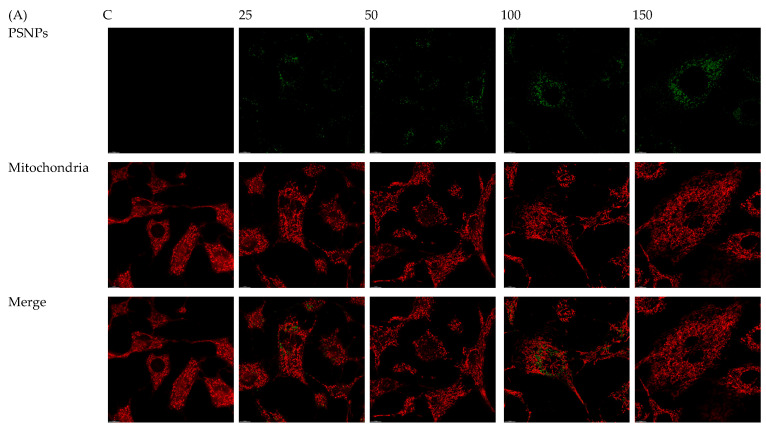

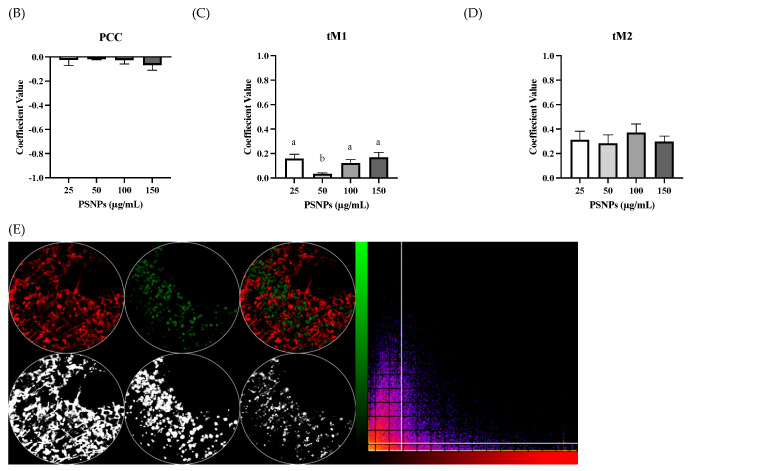
Mitochondrial internalization and colocalization analysis of fluorescent PSNPs in H9c2 cells. Cells were treated with 100 nm fluorescent PSNPs (green) at various concentrations (0, 25, 50, 100, and 150 μg/mL) for 24 h. **(A**) CLSM images of mitochondrial localization of PSNPs in H9c2 cells after exposure to PSNPs (green) for 24 h and with different concentrations. Mitochondria were labeled with MitoTracker Red CMXRos (red). Colocalization between mitochondria and PSNPs was measured by (**B**) PCC and (**C**,**D**) threshold Mander’s correlation coefficients 1 and 2, respectively. (**E**) Colocalization fluorescence intensity scatter plot of 150 μg/mL group generated using Fiji. Data are expressed as mean ± SD (*n* = 6). Statistical analysis was performed using one-way ANOVA followed by Tukey’s post hoc test. Different letters indicate significant differences between groups (*p* < 0.05). C: 0 μg/mL as control group; 25: 25 μg/mL; 50: 50 μg/mL; 100: 100 μg/mL; 150: 150 μg/mL. PSNPs, polystyrene nanoplastics; CLSM, confocal laser scanning microscopy; PCC, Pearson’s correlation coefficient.

Despite efficient cellular uptake, internalized 100 nm fluorescent PSNPs did not translocate into either the nucleus or mitochondria. No colocalization was observed between fluorescent PSNPs and nuclear or mitochondrial fluorescence signals (Figure 4A and Figure 5A). Pearson correlation coefficients were negative or near zero across all treatment groups, indicating minimal or no overlap between fluorescent PSNPs and nuclear or mitochondrial signals (Figure 4B and Figure 5B). Consistently, Mander’s coefficients (tM1 and tM2) revealed very low overlap values, despite a slight increase in nuclear tM1 at 150 μg/mL and a slight decrease in mitochondrial tM1 at 50 μg/mL, confirming the negligible localization of PSNPs within either organelle (Figure 4C,D and Figure 5C,D). Fluorescence scatter plot analysis further demonstrated no correlation between PSNPs and nuclear or mitochondrial signals (Figure 4E,F and Figure 5E).

### 3.5. Mitochondrial Cristae Were Largely Preserved Despite Minor Structural Alterations

**Figure 6:**
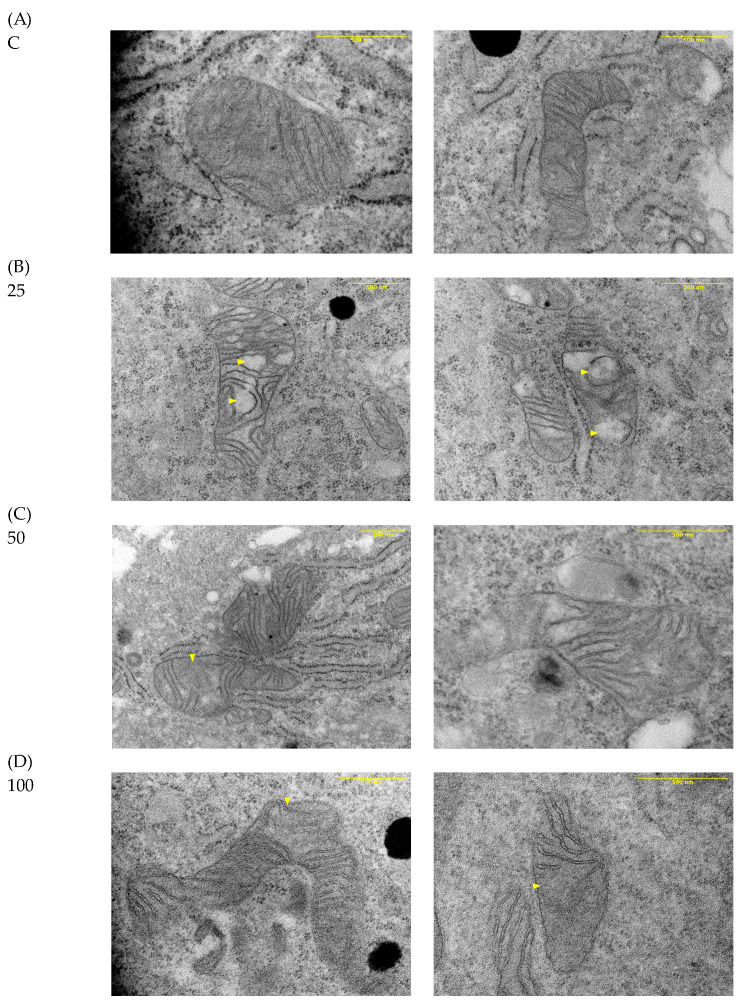

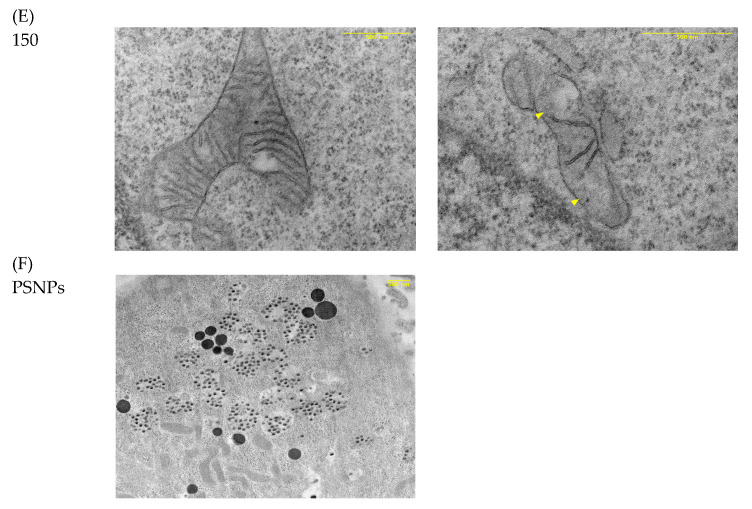
Mitochondrial ultrastructure and cellular distribution of PSNPs in H9c2 cells. Cells were treated with 100 nm PSNPs at various concentrations (0, 25, 50, 100, and 150 μg/mL) for 24 h and observed via TEM. (**A**–**E**) Representative TEM images of mitochondrial ultrastructure. (**A**) Control group (0 μg/mL) and (**B**) 25 μg/mL, (**C**) 50 μg/mL, (**D**) 100 μg/mL, and (**E**) 150 μg/mL PSNP-treated groups (magnification: 30K; scale bar: 500 nm). (**F**) Intracellular distribution and localization of PSNPs in 150 μg/mL treatment group (magnification: 10K; scale bar: 2 μm). C: 0 μg/mL as control group; 25: 25 μg/mL; 50: 50 μg/mL; 100: 100 μg/mL; 150: 150 μg/mL. PSNPs, polystyrene nanoplastics.

H9c2 cells were treated with 0, 25, 50, 100, or 150 μg/mL of 100 nm PSNPs for 24 h and prepared for a TEM analysis of mitochondrial ultrastructure. Figure 6A shows the intracellular localization of PSNPs.

TEM images (Figure 6B–E) show the mitochondrial ultrastructure in PSNP-treated H9c2 cells. In control cells, mitochondria displayed well-defined, densely packed cristae. Following PSNP exposure, mitochondrial swelling accompanied by vacuolization-like structural alterations, widened cristae, and irregular morphology with reduced crista density was observed, as indicated by arrows in the micrographs.

### 3.6. Intracellular and Mitochondrial ROS Production Was Unchanged by PSNP Exposure

**Figure 7:**
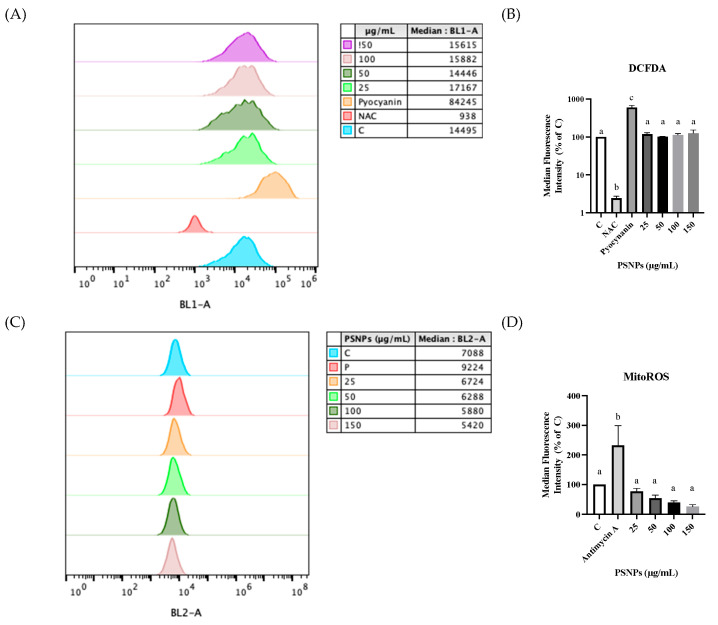
Effects of PSNPs on intracellular and mitochondrial ROS in H9c2 cells. Cells were treated with 100 nm PSNPs at various concentrations (0, 25, 50, 100, and 150 μg/mL) for 24 h. Intracellular and mitochondrial ROS levels were measured using DCFDA and Mitochondrial ROS Assay Kits, respectively, and analyzed by flow cytometry. (**A**) Flow cytometry histogram of intracellular ROS analyzed using FlowJo. (**B**) Intracellular ROS levels presented as MFI. (**C**) Flow cytometry histogram of mitochondrial ROS analyzed using FlowJo. (**D**) Mitochondrial ROS levels presented as MFI. Data are expressed as mean ± SD (*n* = 3). Statistical analysis was performed using one-way ANOVA followed by Tukey’s post hoc test. Different letters indicate significant differences between groups (*p* < 0.05). C: control (0 μg/mL); NAC: N-acetylcysteine (antioxidant control); pyocyanin: positive control for intracellular ROS; antimycin A: positive control for mitochondrial ROS; 25: 25 μg/mL; 50: 50 μg/mL; 100: 100 μg/mL; 150: 150 μg/mL. ROS, reactive oxygen species; DCFDA, 2′,7′-dichlorofluorescin diacetate; MFI, median fluorescence intensity; NAC, N-acetylcysteine.

H9c2 cells were exposed to 0, 25, 50, 100, or 150 μg/mL of 100 nm PSNPs for 24 h. No significant differences were observed among PSNP-treated groups in both intracellular (Figure 7A,B) and mitochondrial ROS levels (Figure 7C,D) compared with the control group. Positive controls (pyocyanin and antimycin A) and negative control (N-acetylcysteine) showed expected responses, confirming assay validity.

### 3.7. Mitochondrial Respiration and Membrane Potential Were Affected by PSNPs

To assess mitochondrial function, mitochondrial membrane potential (ΔΨm) and the OCR were measured using fluorescence-based assays and Seahorse extracellular flux analysis.

**Figure 8:**
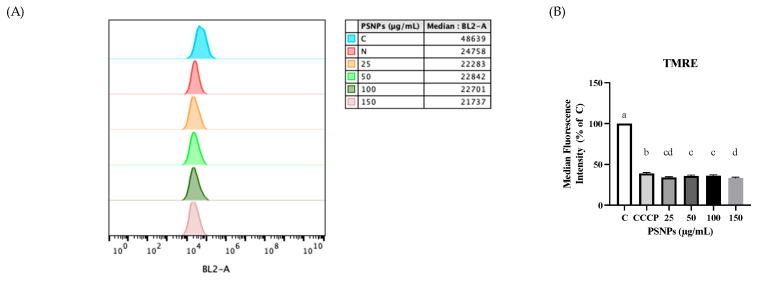
Effects of PSNPs on mitochondrial membrane potential (ΔΨm) in H9c2 cells. Cells were treated with 100 nm PSNPs at various concentrations (0, 25, 50, 100, and 150 μg/mL) for 24 h. Mitochondrial membrane potential was assessed using TMRE Assay Kit and analyzed via flow cytometry. (**A**) Flow cytometry histogram of mitochondrial membrane potential analyzed using FlowJo. (**B**) Quantified mitochondrial membrane potential presented as MFI. Data are expressed as mean ± SD (*n* = 3). Statistical analysis was performed using one-way ANOVA followed by Tukey’s post hoc test. Different letters indicate significant differences between groups (*p* < 0.05). C: 0 μg/mL as control group; CCCP: positive control; 25: 25 μg/mL; 50: 50 μg/mL; 100: 100 μg/mL; 150: 150 μg/mL. CCCP, carbonyl cyanide m-chlorophenyl hydrazone.

H9c2 cells were exposed to 0, 25, 50, 100, or 150 μg/mL of 100 nm PSNPs for 24 h. The PSNP-treated group showed a significant decrease in mitochondrial membrane potential compared to the control group, while there were no significant differences among the PSNP-treated groups (Figure 8A,B).

**Figure 9:**
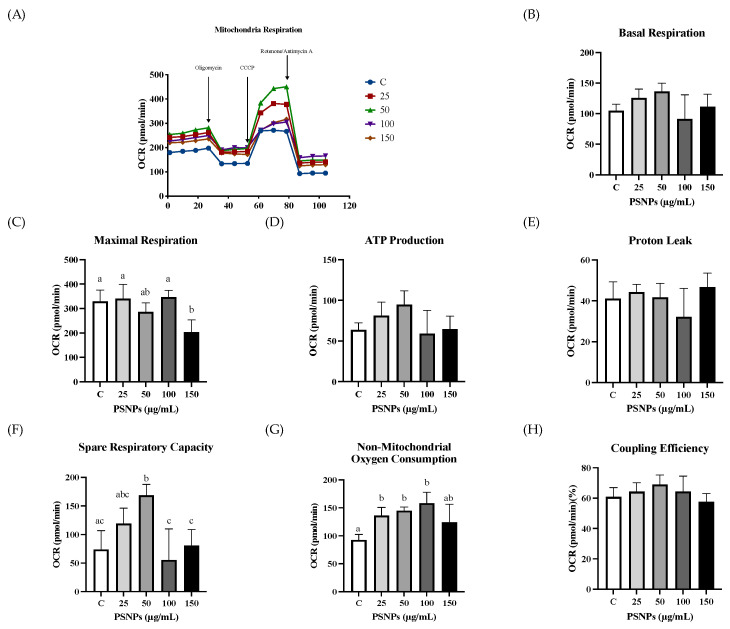
Effects of PSNPs on mitochondrial respiration profiles in H9c2 cells. Cells were treated with 100 nm PSNPs at various concentrations (0, 25, 50, 100, and 150 μg/mL) for 24 h. Mitochondrial respiration was evaluated using Seahorse XF Cell Mito Stress Test Kit on Seahorse XFe24 Extracellular Flux Analyzer. (**A**) OCR profile plot. Quantified individual respiratory parameters are presented as (**B**) basal respiration, (**C**) maximal respiration, (**D**) ATP production, (**E**) proton leak, (**F**) spare respiratory capacity, (**G**) non-mitochondrial oxygen consumption, and (**H**) coupling efficiency. Data are expressed as mean ± SD (*n* = 4). Statistical analysis was performed using one-way ANOVA followed by Tukey’s post hoc test. Different letters indicate significant differences between groups (*p* < 0.05). C: 0 μg/mL as control group; 25: 25 μg/mL; 50: 50 μg/mL; 100: 100 μg/mL; 150: 150 μg/mL. ATP, adenosine triphosphate; OCR, oxygen consumption rate.

Regarding mitochondrial respiration, no significant differences were observed among groups in the basal OCR, ATP production, proton leak, or coupling efficiency (Figure 9B,D,E,H). However, the maximal respiration rate was significantly reduced in the 150 μg/mL group compared with the control group and the 50 μg/mL group (Figure 9C). In contrast, spare respiratory capacity showed a biphasic response, with an increase at 50 μg/mL compared with control but a decrease at 100 and 150 μg/mL relative to the 50 μg/mL group (Figure 9F).

### 3.8. PSNPs Altered Mitochondrial Remodeling and Mitophagy Without Affecting mtDNA

**Figure 10:**
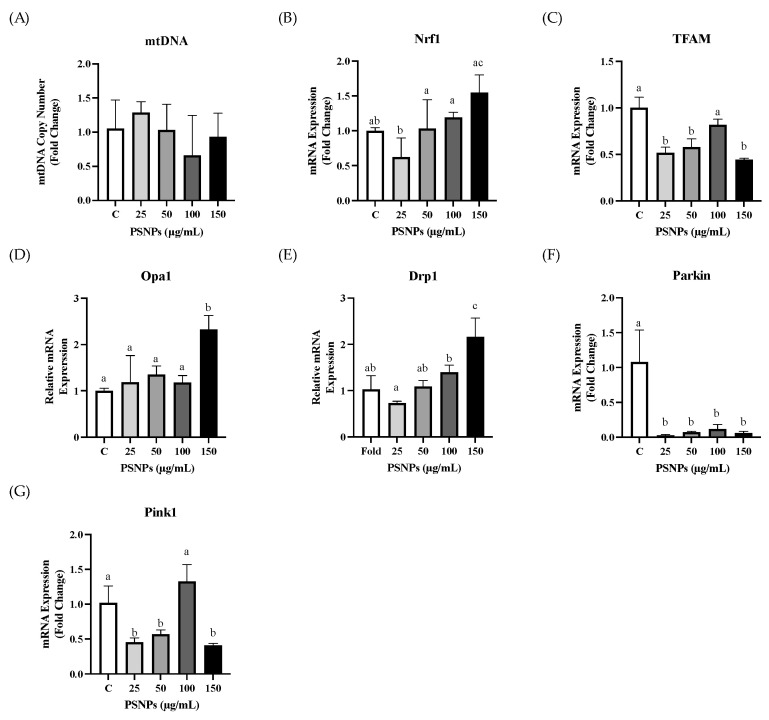
Effects of PSNPs on mitochondrial biogenesis and quality control-related gene expression in H9c2 cells. Cells were treated with 100 nm PSNPs at various concentrations (0, 25, 50, 100, and 150 μg/mL) for 24 h. (**A**) Relative mtDNA copy number. (**B**–**G**) Relative mRNA expression levels of (**B**) *Nrf1*, (**C**) *Tfam*, (**D**) *Opa1*, (**E**) *Drp1*, (**F**) *Parkin*, and (**G**) *Pink1*, normalized to β-*actin* as internal control. Data are expressed as mean ± SD (*n* = 4). Statistical analysis was performed using one-way ANOVA followed by Tukey’s post hoc test. Different letters indicate significant differences between groups (*p* < 0.05). C: 0 μg/mL as control group; 25: 25 μg/mL; 50: 50 μg/mL; 100: 100 μg/mL; 150: 150 μg/mL. mtDNA, mitochondrial DNA; q-PCR, quantitative PCR; Nrf1, nuclear respiratory factor 1; Tfam, mitochondrial transcription factor A; Opa1, optic atrophy 1; Drp1, dynamin-related protein 1; Pink1, PTEN-induced putative kinase 1; Parkin, E3 ubiquitin protein ligase.

H9c2 cells were exposed to 0, 25, 50, 100, or 150 μg/mL of 100 nm PSNPs for 24 h, and mitochondrial biogenesis, dynamics, and mitophagy were assessed by measuring mRNA levels. Compared with the control group, nuclear respiratory factor 1 (*Nrf1*) mRNA expression showed no significant differences, whereas mitochondrial transcription factor A (*Tfam*) mRNA was significantly decreased in PSNP-treated cells (Figure 10B,C).

Regarding mitochondrial dynamics, the mRNA expression levels of optic atrophy 1 (*Opa1*), a fusion-related gene, and dynamin-related protein 1 (*Drp1*), a fission-related gene, were both significantly increased in the 150 μg/mL group, indicating enhanced mitochondrial remodeling (Figure 10D,E). Mitophagy-related gene expression was also reduced after PSNP exposure: PTEN-induced kinase 1 (*Pink1*) decreased in the 25, 50, and 150 μg/mL groups, and *Parkin* decreased in all treated groups (Figure 10F,G), while mtDNA copy number remained unchanged (Figure 10A). Despite the reduction in mitochondrial membrane potential, mitophagy-related gene expression was decreased rather than increased.

### 3.9. Basal Glycolytic Activity and Cell Survival Remained Unaffected by PSNPs

To further explore the impact of PSNPs on H9c2 cellular homeostasis, we evaluated cellular energy metabolism, including glycolysis-related parameters (lactate production and ECAR), as well as apoptosis.

**Figure 11:**
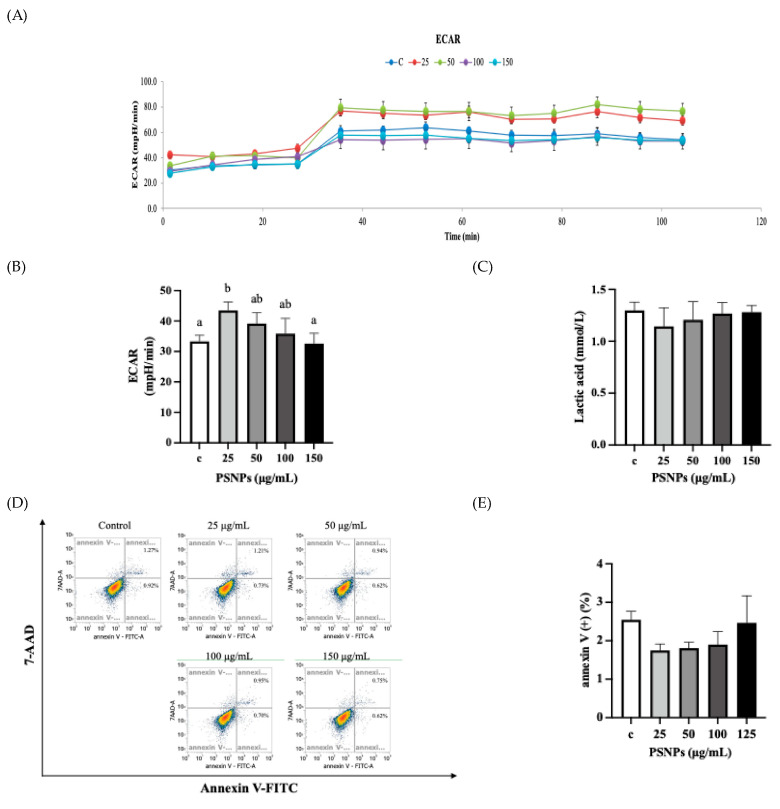
Evaluation of glycolytic compensation and cell survival following PSNP exposure in H9c2 cells. Cells were treated with 100 nm PSNPs at various concentrations (0, 25, 50, 100, and 150 μg/mL) for 24 h. (**A**) ECAR kinetic profile plot over time. (**B**) Quantified basal ECAR, calculated as average of measurements obtained prior to first oligomycin injection. (**C**) Glycolytic activity assessed based on extracellular lactate production. (**D**) Representative flow cytometry quadrant plots showing apoptotic cell populations analyzed via Annexin V/7-AAD staining. (**E**) Quantified total apoptotic rate, presented as percentage of apoptotic cells. Data are expressed as mean ± SD (*n* = 4). Statistical analysis was performed using one-way ANOVA followed by Tukey’s post hoc test. Different letters indicate significant differences between groups (*p* < 0.05). C, control group (0 μg/mL); 25, 25 μg/mL; 50, 50 μg/mL; 100, 100 μg/mL; 150, 150 μg/mL. ECAR, extracellular acidification rate; PSNPs, polystyrene nanoplastics; 7-AAD, 7-aminoactinomycin D.

H9c2 cells were exposed to 0, 25, 50, 100, or 150 μg/mL of 100 nm PSNPs for 24 h. PSNP exposure did not markedly alter ECAR or lactic acid production, an indirect indicator of glycolytic metabolism, suggesting limited effects on basal cellular energy metabolism (Figure 11A,B). Although a significant difference was observed between the control and 25 μg/mL groups, no clear dose-dependent trend was detected. In addition, no significant apoptosis was observed under these conditions (Figure 11C,D). These findings suggest that PSNP exposure exerted minimal effects on basal glycolytic activity and cell survival in H9c2 cells.

## 4. Discussion

In this study, H9c2 cells were treated with 100 nm PSNPs at concentrations ranging from 0 to 150 μg/mL for 24 h. Under these experimental conditions, no significant effects on cell viability were observed. Previous studies have suggested that PSNPs may induce a decrease in mitochondrial membrane potential, increase ROS production, and trigger apoptosis by affecting endoplasmic reticulum Ca^2+^ dynamics [24]. However, other reports have indicated that while PSNPs can alter mitochondrial morphology, these effects are not necessarily accompanied by increased oxidative stress or reduced cell viability [25]. Overall, the effects of PSNPs on cytotoxicity and oxidative stress may depend on cell type, particle size, and surface properties [26,27,28], which may explain why no decrease in viability was observed under the conditions of this study, consistent with the previous literature [29].

The results of this study demonstrated that 100 nm PSNPs were internalized by H9c2 cells. The use of confocal microscopy combined with colocalization analysis minimized potential visual bias from fluorescent dyes. The findings indicated that PSNPs accumulated in the cytoplasm and lysosomes in a time- and dose-dependent manner, but no entry into the nucleus or mitochondria was observed. PSNPs of different sizes may utilize distinct internalization mechanisms. In RBL-2H3 basophilic leukemia cells, 50 nm PSNPs are likely internalized via clathrin- or caveola-mediated endocytosis, whereas 500 nm PSNPs tend to enter cells through macropinocytosis [30]. Previous studies have also reported that smaller PSNPs are generally more readily internalized and accumulate in the cytoplasm [2]. Therefore, once internalized, PSNPs may further influence multiple intracellular organelles and metabolic functions, and their potential effects on cellular energy metabolism warrant further investigation.

After exposure to PSNPs, mitochondria exhibited widened crista spacing and vacuole-like structural changes at 100 and 150 μg/mL, accompanied by irregular shapes, reduced crista numbers, and elongated morphology, thus indicating potential alterations in mitochondrial ultrastructure. In Leydig cells, treatment with 20 nm PSNPs at 0–150 μg/mL for 24 h resulted in the loss of cristae, vacuole-like structural changes, and enlarged, swollen mitochondria [31]. In a mouse study, the administration of 70 nm PSNPs at 80 μg/kg for 26 weeks led to mitochondrial crista disruption, endoplasmic reticulum dilation, crista loss, and swelling in hepatocytes [32]. The cristae of the mitochondrial inner membrane are the primary sites for oxidative phosphorylation and ATP production, and their formation and maintenance are regulated by the mitochondrial contact site and cristae organizing system (MICOS) complex, Opa1, and ATP synthase [33].

Previous studies have suggested that PSNPs may affect mitochondrial function through multiple mechanisms, including NOX4-mediated ROS feedback loops [34], ER–mitochondrial calcium dysregulation [35], and membrane interactions associated with the disruption of the mitochondrial ETC [26], ultimately contributing to oxidative stress and mitochondrial dysfunction. Under stress or aging, cristae can become loosened and expanded, leading to decreased respiratory capacity, reduced ATP production, increased ROS generation, and a higher risk of cell death [36]. Consistent with this, we further evaluated intracellular and mitochondrial ROS levels to assess oxidative stress status following PSNP exposure.

Previous studies have suggested that PSNPs increase mitochondrial oxidative stress and decrease mitochondrial membrane potential and ATP production [37]. It has been proposed that PSNPs may elevate mitochondrial oxidative stress by disrupting calcium homeostasis, reducing mitochondrial membrane potential, and impairing antioxidant enzyme activity [38,39]. However, a study has also reported that long-term exposure to PSNPs does not induce an increase in intracellular ROS levels [25]. Taken together, these findings suggest that PSNP-induced oxidative stress and cytotoxicity may vary depending on particle size and surface properties [26,40]. In particular, smaller particles or positively charged PSNPs are more likely to disrupt cellular structures and induce oxidative stress, thereby exhibiting higher cytotoxic potential.

Changes in the mitochondrial structure may represent an early cellular response to PSNP exposure; therefore, this study first investigated whether mitochondrial dynamics are involved in the cellular response to PSNPs and analyzed the expression of genes related to mitochondrial fusion and fission. The results showed that PSNPs may modulate mitochondrial dynamics to cope with their uptake, while the overall mitochondrial mass remained unaffected. Mitochondrial dynamics-related genes *Opa1* and *Drp1* were significantly upregulated after high-dose PSNP treatment, potentially reflecting a cellular response to mitochondrial stress. Previous studies have reported that PSNP/PSMP exposure can disrupt the balance of mitochondrial fusion and fission, with most indicating the activation of mitochondrial dynamic regulation through an increase in fission-related proteins (Drp1, Fis1) and changes in fusion proteins (Mfn1/2, Opa1) [41,42,43,44]. Consistent with these trends, this study also observed the simultaneous upregulation of *Opa1* and *Drp1*, suggesting that PSNP exposure may enhance both mitochondrial fusion and fission, reflecting the dynamic remodeling of mitochondria in response to cellular stress.

Besides the changes in mitochondrial dynamics, whether total mitochondrial content or biogenesis was affected requires further investigation. In this study, PSNP exposure reduced *Tfam* mRNA expression without causing a significant change in mtDNA copy number. This observation is consistent with previous findings indicating that alterations in *Tfam* expression do not necessarily translate into corresponding changes in mitochondrial biogenesis or mtDNA content under certain conditions [45]. Reduced *Tfam* mRNA expression without altered mtDNA copy number indicates that PSNP exposure primarily affects transcriptional regulation rather than mitochondrial content.

Membrane potential depolarization is generally considered a necessary condition for initiating Pink1/Parkin-dependent mitophagy [46]. However, in the present study, exposure to PSNPs suppressed the mRNA expression of *Pink1* and *Parkin*. This discrepancy suggests that PSNPs may impair the mitochondrial quality control mechanism rather than triggering protective mitophagy, leading to the accumulation of dysfunctional mitochondria. Consistent with prior studies, our findings indicate that a decrease in membrane potential is necessary but not sufficient to initiate mitophagy.

The results of the present study differ from several reports suggesting that PSNPs can activate the Pink1/Parkin pathway and promote mitophagy [42,44,47]. This may indicate that the effects of PSNPs on mitochondria may depend on particle size, exposure dose, cell type, or treatment duration [27,48,49]. It may also reflect that under higher stress conditions, mitochondrial quality control mechanisms are impaired, resulting in the ineffective clearance of damaged mitochondria [50]. Overall, PSNPs may lead to increased mitochondrial dynamics but an imbalance in mitophagy, ultimately affecting mitochondrial stability and cellular function.

Since mitochondria are the primary source of cellular ATP, the impairment of oxidative phosphorylation or suppressed mitochondrial respiration can lead cells to increase glycolysis to maintain ATP supply. Such compensatory glycolysis is often reflected by an elevated ECAR and lactate production [51]. Therefore, this study further measured lactate production and the ECAR to assess potential metabolic adjustments. The results showed no marked changes in either parameter across most treatment groups, indicating that PSNP-induced mitochondrial damage did not trigger compensatory glycolysis or major metabolic shifts. Despite the lack of evident effects on basal metabolic function, TEM observations revealed vacuole-like structural changes in the mitochondrial inner membrane, suggesting that PSNPs altered the mitochondrial ultrastructure. This may represent early or mild structural damage that does not compromise overall cellular energy metabolism, as reflected by the unchanged basal OCR, ECAR, and lactate production observed in this study. Concurrently, PSNP exposure significantly decreased maximal respiration, while spare respiratory capacity appeared to increase compensatorily at 25–100 μg/mL but decreased and returned to near baseline at 100 and 150 μg/mL. These findings suggest that mitochondrial functional flexibility under stress may be temporarily enhanced at low PSNP concentrations but progressively lost at higher concentrations. Overall, the effects of PSNPs likely reflect an early-stage mitochondrial stress state, where cells can still maintain basal energy homeostasis, but their respiratory capacity and adaptive flexibility under higher energy demand are potentially compromised.

Several methodological considerations should be noted when interpreting the present results. H9c2 cells were used as an in vitro cardiomyoblast model and may not fully recapitulate the physiology of mature cardiomyocytes. In addition, while biological replicates were included across experiments, some assays (e.g., uptake and mitochondrial membrane potential) were performed with limited sample size (*n* = 3) due to technical constraints. Finally, all experiments were conducted at a single time point (24 h), which does not allow for the evaluation of the temporal dynamics of the observed effects. Future studies incorporating multiple time points, increased replication, and in vivo models will be important to further validate and extend these findings.

In summary, 100 nm PSNPs showed no evidence of mitochondrial localization within 24 h and induced no statistically significant acute cytotoxicity or basal metabolic dysfunction in H9c2 cells. However, intracellular accumulation was observed, accompanied by alterations in mitochondrial ultrastructure, reduced mitochondrial membrane potential, and the modulation of genes related to mitochondrial quality control. In particular, the loosening of cristae and vacuole-like structural changes indicate that the inner mitochondrial membrane may have undergone early structural impairment.

Lactate production and the ECAR remained largely unchanged, suggesting that a marked glycolytic compensatory response was not triggered under these conditions. Nevertheless, the decline in maximal respiratory capacity indicates reduced mitochondrial performance under high energy demand. Together, these findings suggest that PSNP-induced mitochondrial alterations represent an early stage of functional imbalance. At this stage, overall cell viability and basal metabolism are maintained, yet the adaptive capacity of cardiomyoblasts under stress conditions may be compromised.

Overall, the present study demonstrates that even in the absence of apparent cytotoxicity, PSNP exposure can perturb mitochondrial structure and function in cardiomyoblasts, highlighting a potential risk to cardiovascular health and emphasizing the need for further investigation into its long-term effects.

## 5. Conclusions

In conclusion, 100 nm PSNPs showed no evidence of mitochondrial localization and induced no statistically significant cytotoxicity within 24 h. However, intracellular accumulation was associated with alterations in mitochondrial ultrastructure, reduced membrane potential, the modulation of mitochondrial quality control-related genes, and decreased maximal respiratory capacity, while basal metabolism remained unchanged. These findings indicate that PSNP exposure induces early mitochondrial structural and functional disturbances without acute cell death. Although overall viability is preserved, mitochondrial adaptive capacity under stress conditions may be compromised. Collectively, this study suggests that subclinical mitochondrial impairment may represent an early event in PSNP-associated cardiotoxicity and warrants further investigation into its long-term implications using animal models.

## Data Availability

The original contributions presented in this study are included in the article, and further inquiries can be directed to the corresponding author.

## Supplementary Materials

The following supporting information can be downloaded at https://www.mdpi.com/article/10.3390/antiox15070801/s1, Table S1: Primer sequences for determination of gene expression levels.

## Abbreviations

The following abbreviations are used in this manuscript:

7-AAD	7-aminoactinomycin D
ATP	Adenosine triphosphate
CVDs	Cardiovascular diseases
DCFDA	2′,7′- dichlorofluorescin diacetate
DLS	Dynamic light scattering
DMEM	Dulbecco’s modified Eagle’s medium
DRP1	Dynamin-related protein 1
ECAR	Extracellular acidification rate
EDTA	Ethylenediaminetetraacetic acid
ELS	Electrophoretic light scattering
ETC	Electron transport chain
FBS	Fetal bovine serum
FITC	Fluorescein isothiocyanate
FSC	Forward scatter
HBSS	Hank’s Balanced Salt Solution
MFI	Mean fluorescence intensity
MICOS	Mitochondrial contact site and cristae organizing system
MPs	Microplastics
MQC	Mitochondria quality control
mtDNA	Mitochondrial DNA
MTT assay	3-(4,5-dimethylthiazol-2-yl)-2,5-diphenyltetrazolium bromide assay
NPs	Nanoplastics
Nrf1	Nuclear respiratory factor 1
OCR	Oxygen consumption rate
Opa1	Optic atrophy 1
PA	Polyamide
PBS	Phosphate-buffered saline
PCC	Pearson’s correlation coefficient
PE	Polyethylene
PET	Polyethylene terephthalate
Pink1	PTEN-induced kinase 1
PP	Polypropylene
PSNPs	Polystyrene nanoplastics
PS	Polystyrene
PU	Polyurethane
PVC	Polyvinyl chloride
q-PCR	Quantitative polymerase chain reaction
ROS	Reactive oxygen species
SD	Standard deviation
SSC	Side scatter
TEM	Transmission electron microscopy
Tfam	Transcription factor A
TMRE	Tetramethylrhodamine ethyl ester
UNEA	United Nations Environment Assembly
