# Supplementary material for: Polystyrene Nanoplastics Induce Early Mitochondrial Dysfunction in H9c2 Cardiomyoblasts Without Substantial Cell Damage
Source: Antioxidants (Basel). 2026 Jun 26;15(7):801. doi: 10.3390/antiox15070801 (PMC13405344; doi:10.3390/antiox15070801)
Supplement: Supplementary file 1 [file antioxidants-15-00801-s001.zip › antioxidants-4358771-supplementary.pdf]

Supplementary Materials

Table S1. Primer sequences for determination of gene expression levels.

Gene	Accession numbers	Sequences (5' to 3')	Reference
TFAM	NM_031326.2	F: TGTCATTGGGATTGGGCACA<br>R: AGATGCACGCACAGTCTTGA	[19]
Nrfl	NM_001100708.1	F: GCAACAGGGAAGAAACGG<br>R: GTGTCTGCTGTCTCTTTCGGATA	[20]
Opal	NM_001433910.1	F: GCATCTGGTTTCCCGAAGTA<br>R: GAGCCTTGCAGCTAACCTTG	[20]
Drp1	NM_001437685.1	F: GTAGGTGATCAGCCCAAGGA<br>R: CATCTGGATCTACCTCTCTGGAA	[20]
Pink 1	NM_001438119.1	F: ACTACCTATGCCCATCCATCTA<br>R: CTCGGTGACAGCTAAGTCAT	[21]
Parkin	NM_020093.1	F: CCGGTGACCATGATAGTGTTT<br>R: CACTTCCTTGAGCTGGAAGAT	[22]
ND-1	NC_086026.1	F: CCCTAAAACCCGCCACATCT<br>R: GAGCGATGGTGAGAGCTAAG	[18]
β-Actin	NM_031144.3	F: ATGGTGGGTATGGGTCAGAA<br>R: CTTTTCACGGTTGGCCTTAG	[20]

18. Li Y, Chen H, Yang Q, Wan L, Zhao J, Wu Y, et al. Increased Drp1 promotes autophagy and ESCC progression by mtDNA stress mediated cGAS-STING pathway. J Exp Clin Cancer Res. 2022;41(1):76.

19. Gul R, Okla M, Mahmood A, Nawaz S, Fallata A, Bazighifan A, et al. Comparison of the Protective Effects of Nebivolol and Metoprolol against LPS-Induced Injury in H9c2 Cardiomyoblasts. Curr Issues Mol Biol. 2023;45(11):9316-27.

20. Gunaydin Akyildiz A, Boran T, Jannuzzi AT, Alpertunga B. Mitochondrial dynamics imbalance and mitochondrial dysfunction contribute to the molecular cardiotoxic effects of lenvatinib. Toxicol Appl Pharmacol. 2021;423:115577.

21. Wang SH, Zhu XL, Wang F, Chen SX, Chen ZT, Qiu Q, et al. LncRNA H19 governs mitophagy and restores mitochondrial respiration in the heart through Pink1/Parkin signaling during obesity. Cell Death Dis. 2021;12(6):557.

22. Yu J, Li Y, Liu X, Ma Z, Michael S, Orgah JO, et al. Mitochondrial dynamics modulation as a critical contribution for Shenmai injection in attenuating hypoxia/reoxygenation injury. J Ethnopharmacol. 2019;237:9-19.
